# Quality assurance guidelines for superficial hyperthermia clinical trials: II. Technical requirements for heating devices

**DOI:** 10.1007/s00066-017-1106-0

**Published:** 2017-03-01

**Authors:** Hana Dobšíček Trefná, Johannes Crezee, Manfred Schmidt, Dietmar Marder, Ulf Lamprecht, Michael Ehmann, Jacek Nadobny, Josefin Hartmann, Nicolleta Lomax, Sultan Abdel-Rahman, Sergio Curto, Akke Bakker, Mark D. Hurwitz, Chris J. Diederich, Paul R. Stauffer, Gerard C. Van Rhoon

**Affiliations:** 1 Signals and Systems, Chalmers University of Technology, Gothenburg, Sweden; 2 Radiotherapy, AMC, Amsterdam, The Netherlands; 3 Radiotherapy Clinics, Universitatsklinikum Erlangen, Erlangen, Germany; 4 Kantonsspital Aarau, Aarau, Switzerland; 5 Radiation Oncology, University Hospital Tuebingen, Tuebingen, Germany; 6 Radiation Oncology, University Medical Centre Mannheim, Mannheim, Germany; 7 Klinik für Radioonkologie und Strahlentherapie, Campus Virchow Klinikum, Charité Universitatsmedizin Berlin, Berlin, Germany; 8 Department of Internal Medicine III, Ludwig Maximilians University of Munich, Munich, Germany; 9 Radiation Oncology, Erasmus MC Daniel den Hoed Cancer Center, Rotterdam, The Netherlands; 10 Department of Radiation Oncology, Thomas Jefferson University, Philadelphia, PA USA; 11 Department of Radiation Oncology, UCSF, San Francisco, CA USA

**Keywords:** Quality assurance, Hyperthermia, superficial, Applicator, Water bolus, Phantoms, Heating criteria, Qualitätssicherung, Hyperthermie, lokale, Applikator, Wasserbolus, Phantome, Heizkriterien

## Abstract

Quality assurance (QA) guidelines are essential to provide uniform execution of clinical trials with uniform quality hyperthermia treatments. This document outlines the requirements for appropriate QA of all current superficial heating equipment including electromagnetic (radiative and capacitive), ultrasound, and infrared heating techniques. Detailed instructions are provided how to characterize and document the performance of these hyperthermia applicators in order to apply reproducible hyperthermia treatments of uniform high quality. Earlier documents used specific absorption rate (SAR) to define and characterize applicator performance. In these QA guidelines, temperature rise is the leading parameter for characterization of applicator performance. The intention of this approach is that characterization can be achieved with affordable equipment and easy-to-implement procedures. These characteristics are essential to establish for each individual applicator the specific maximum size and depth of tumors that can be heated adequately. The guidelines in this document are supplemented with a second set of guidelines focusing on the clinical application. Both sets of guidelines were developed by the European Society for Hyperthermic Oncology (ESHO) Technical Committee with participation of senior Society of Thermal Medicine (STM) members and members of the Atzelsberg Circle.

## Zusammenfassung

Um eine einheitliche Durchführung klinischer Studien in der Hyperthermie zu gewährleisten, sind Leitlinien zur Qualitätssicherung (QA) unerlässlich. Dieses Dokument enthält die Anforderungen zur QA für alle aktuellen Therapiegeräte zur lokalen Hyperthermie, inklusive elektromagnetischer Systeme (radiativ und kapazitiv), Ultraschall- und Infrarottechnik. In detaillierten Anleitungen wird erklärt, wie die Leistungscharakteristik der Hyperthermieapplikatoren zu beschreiben und zu dokumentieren ist, um reproduzierbare, einheitliche und qualitativ hochwertige Hyperthermiebehandlungen zu gewährleisten. Im Gegensatz zu früheren Leitlinien wird anstelle der spezifischen Absorptionsrate (SAR) jetzt der Temperaturanstieg als maßgeblicher Parameter für die Beschreibung der Leistungscharakteristik von Hyperthermieapplikatoren verwendet. Grund für diesen Ansatz ist eine möglichst einfache und kostengünstige Leistungsbeschreibung. Diese ist notwendig, um für jeden einzelnen Applikator die maximale Tumorgröße und -tiefe zu bestimmen, die adäquat überwärmt werden kann. Dieses Dokument wird durch die Leitlinie zur klinischen Durchführung der lokalen Hyperthermie vervollständigt. Beide Teile der Leitlinie wurden vom Technischen Komitee der European Society for Hyperthermic Oncology (ESHO) in Zusammenarbeit mit leitenden Mitgliedern der Society of Thermal Medicine (STM) und Mitgliedern des Atzelsberger Kreises erarbeitet.

## Introduction

These quality assurance (QA) guidelines for the application of superficial hyperthermia clinical trials were developed at the request of the Atzelsberg Circle for Clinical Hyperthermia of the interdisciplinary working group of hyperthermia “Interdisziplinäre Arbeitsgruppe Hyperthermie” (IAH) [1] of the German Cancer Society (“Deutsche Krebsgesellschaft”) to the European Society for Hyperthermic Oncology (ESHO) [2]. ESHO delegated this task to the ESHO technical committee (TC), who formulated the guidelines with participation of experienced members of the Society for Thermal Medicine (STM) [3]. In addition, the manufacturers providing equipment for superficial hyperthermia were invited to provide their feedback on the QA guidelines during public sessions at the 2014 and 2015 annual meetings of ESHO or alternatively by personal communication.

The QA guidelines seek to establish a minimum level of treatment quality in hyperthermia treatments delivered in all multi-institutional studies initiated by the Atzelsberg Circle or under the auspices of the ESHO. The goal of this effort is to establish QA guidelines for the application of superficial hyperthermia, similar to the QA guidelines for administration of deep hyperthermia defined earlier [4, 5] and as a long awaited follow up to previous superficial hyperthermia QA guidelines provided by the Radiation Therapy Oncology Group and ESHO [6–8].

These QA guidelines for the application of superficial hyperthermia clinical trials consists of two parts:

- Part I [9] provides detailed instructions on treatment documentation, defines a good hyperthermia treatment, and identifies the clinical conditions where a certain hyperthermia system can or cannot adequately heat the tumor volume.
- Part II, i. e., this document, provides quality assurance requirements for heating equipment as well as detailed instructions how to characterize and document the performance of hyperthermia devices in order to apply reproducible, uniform, and high quality hyperthermia treatments. These characteristics help to establish the maximum size and depth of tumors that can be heated adequately.

Implementation of these QA guidelines should facilitate correct assessment of whether a tumor can or cannot be heated with the specific heating device(s) available in a hyperthermia center and, thus, enable a decision as to whether a patient is or is not eligible to participate in a clinical study.

Many different systems are used to apply superficial hyperthermia, either built commercially or in academic research laboratories. Each system has unique characteristics and advantages (as summarized in Appendix A), which may result in a large heterogeneity in the quality of applied hyperthermia treatments between various hyperthermia centers.

To assure proper performance of a superficial hyperthermia applicator, the spatial thermal pattern should be evaluated under well-controlled conditions prior to the first clinical use of the applicator and at regular intervals as part of a clinical QA program. Due to time constraints in the clinical workflow and the large diversity of superficial heating systems, which include electromagnetic (EM), radiofrequency (RF), ultrasound (US), and infrared (IR) technologies, these new QA guidelines require characterization of the heating performance of hyperthermia equipment with temperature (rise) simulations and measurements in homogeneous muscle tissue-equivalent phantoms. This is a more pragmatic approach than that used in previous guidelines/recommendations, which were based on power deposition patterns quantified in W/kg and described as Specific Absorption Rate (SAR) distributions. For homogenous nonperfused phantom models, the SAR and temperature rise in a defined period of time are directly related, since no convective heat losses are present [10].

## Definitions and characteristic features of a superficial hyperthermia system

### Applicators

Superficial applicators, used today, consist of

- External EM antennas or waveguides,
- External EM capacitive electrodes (“capacitive” RF systems),
- External US transducers (US systems), and
- External noncontacting IR heating systems.

In general, these devices deposit energy to heat a limited volume of tissue close to the heating device. A brief summary of the operating principles of various systems for heating superficial tissue is given in Part I of the QA guidelines [9], while more detailed equipment options are given in Appendix A. Further information can be found in reviews [11–14].

#### Superficial hyperthermia applicator terminology

- *Single applicator* **:** A single radiating aperture connected to a power amplifier having independent control of output power from 0–100%. The applicator can be an EM radiator [16–24], US transducer [14], IR lamp [15], or the active electrode of a capacitive system [52, 53].
- *Multi-element applicator array:* To produce effective heating of large area tumors, several single applicators can be combined into larger arrays with separate power control of each element to enable 2D power steering [25, 29, 34, 36]. Examples include patient-customized arrays of separately placed applicators such as 2–6 lucite cone applicator (LCA) [18] or two adjacent contact flexible microstrip applicator (CFMA) [19].
- *Multi-element array applicator:* Alternatively a heat applicator can be constructed with a fixed spatial array of independently controlled heating elements, such as spiral microstrip [26–28], conformal microwave array (CMA) [30–33], Microtherm 1000 planar array [35] applicators, or a fixed array of ultrasound apertures, such as four and sixteen element devices [60, 61].

In order to generate sufficiently uniform and repeatable heating, special care must be devoted to interapplicator spacing and noncoherent phase excitation in order to minimize cross-coupling and phase interaction between adjacent applicators and to achieve a contiguous SAR/temperature distribution above the 50% iso-SAR or 50% isotemperature level across the tissue target between applicator elements.

Single applicators and multi-element applicators should fulfill the following criteria or be characterized as follows:

- Every single radiating aperture should be capable of producing effective superficial HT under the aperture (Section *Test conditions*).
- Every radiating element of an applicator array or array applicator must be powered noncoherently in order to avoid cross-coupling effects between the elements.
- Multiple coherently radiating elements may be considered as a single independent radiating element if a common wave front is created parallel to the surface.
- The therapeutic region of an array applicator or an applicator array is defined analogously to the definition of therapeutic region for a single applicator.

It is strongly recommended to include thermometry measurement points underneath each independently powered element of an array applicator or applicator array.

### Technical considerations for the water bolus

The function of the temperature-controlled water-circulating bolus is to couple electromagnetic or ultrasound energy into the patient and to control the skin surface temperature. IR systems generally operate without a water bolus and can achieve some control of skin surface temperatures through forced convection of air.

#### General requirements for the water bolus

An optimal contact area of the water bolus with the skin of the target surface requires that the bolus is sufficiently large to smoothly follow the skin contour [37–40] and that the bolus extends beyond the radiating aperture. This provides better coupling of EM/US field to tissue without distorting the radiation pattern; at the same time it puts greater demands on reproducible alignment of the applicator relative to the tumor margin. An adequate bolus design is required since the dimensions and temperature of the water bolus significantly affect the applicator power deposition (SAR) pattern, thermal effective field size (TEFS), and thermal depth profiles. For EM radiative applicators, the bolus must be filled with deionized water, whereas for capacitive heating saline bolus is generally preferred. For US transducers, the water must be degassed as well. In all cases, the water must be circulated through the bolus with a circulation pump and the temperature controlled within the range of 15^1^ to 45 °C. A bubble trap, impurity filter and a flow indicator is recommended to be included in the circulation system. The input and output flow connectors of the water bolus are located on opposite sides. Large applicator arrays may require dual-input–dual-output ports in order to maintain acceptable temperature homogeneity (<1 °C variation) across the bolus surface [39–41]. The water bolus can be filled with low density semirigid porous foam, plastic spacers, or thin rubber pins to avoid collapse of the bolus while pressed against the skin surface.

#### Specific features of the water bolus

Some manufacturers provide a water bolus that is an integral part of the applicator with a rigid plastic frame that mounts to the applicator aperture [19, 22, 27–29, 33, 35]. While this makes bolus position more reproducible relative to the applicator, the consequence of a fixed-frame set-up is often a convex bolus shape with reduced contact area between water bolus and skin that is smaller than the radiating aperture. This may cause a localized E‑field discontinuity and associated high tissue temperature rise near the sharp transition in EM coupling at the water bolus–air interface if that occurs under a high field region of the radiating aperture [41]. Ultrasound systems require deionized and degassed water, with the degree of degassing required inversely proportional to frequency; this is most critical for systems operating at 0.5–1.5 MHz where a dissolved oxygen content below 0.1 ppm is required to avoid lossy propagation and outgassing of air from the coupling bolus during ultrasound transmission. In EM capacitive heating systems, saline (0.1–1.5%) is generally used [43, 44] to improve impedance matching between electrodes and muscle tissue, as well as to spread RF currents over the contact area and thereby reduce skin burns at the edge of the electrode.

#### Guidelines for proper design and evaluation of the water bolus

- Size: EM-radiative applicators: the water bolus should extend at least beyond the perimeter of the applicator aperture and ideally at least 2–5 cm outside the perimeter. Phantom experiments of the applicator with various bolus sizes are recommended.
  EM-capacitive systems: the TEFS is highly dependent on the size of the contact area of the coupling bolus and tissue. Especially at the edge of the bolus, care must be taken to provide smooth transitions at the bolus–skin contact at the peripheral rim of the water bolus [42–44].
- Thickness: For EM-radiative applicators the optimal water bolus thickness depends on type of applicator, its dimensions as well as target depth [41] and the thickness should not exceed critical values to avoid oscillation modes in the bolus layer [45, 46]. These modes occur for larger sized applicators at frequencies above 100 MHz [47] and also for suboptimal contact between bolus and applicator [20].
  EM-capacitive systems utilize a frequency below 100 MHz. They require a minimum water bolus thickness depending on applicator size to minimize the effect of concentrated current at the edge of the metal electrodes. Their performance is rather insensitive to a variation in water bolus thickness provided the minimum thickness requirement is satisfied [43, 44].
  For ultrasound applicators with properly degassed water bolus, thickness has negligible effect on energy propagation (losses) since the attenuation in water is very low, but may affect beam pattern shape and position of the focal zone depending on the device and configuration. Phantom experiments or direct SAR or intensity measurements must be designed specific for each applicator with various bolus thicknesses in the clinically relevant range.
- Variations in bolus thickness (±50%) are inevitable in clinical conditions due to the irregular skin surface contour. In order to assure that users know how to respond effectively to patient complaints and to unexpected temperature heterogeneity, the impact of these variations on field homogeneity and effective field size should be assessed experimentally or by simulations.
- Good contact between bolus and phantom: Wet gauze can be used to improve heat transfer at the bolus–skin interface for EM devices [41] but proper care must be taken to ensure that no air gaps occur in the interface [48]. Air bubbles and air gaps need to be removed with acoustic gel or water coupling for ultrasound devices.
- The water temperature has a profound impact on the effective penetration depth: The use of cold water will increase the effective penetration depth of the maximum temperature and the therapeutic extent up to 1–2 cm [41, 49]. Experimental evaluation with phantom or pennes bioheat equation(PBHE)-based models to estimate the actual penetration depth is therefore recommended for specific energy modalities and bolus configurations. These models can also be applied for estimating the impact of water bolus dimensions and flow.

## Characterization of heating properties

### Temperature rise criteria for adequate heating

ESHO-TC defines a heating device to be adequate/appropriate if a temperature rise (TR) of at least 6 °C above the starting temperature can be achieved in 6 min at 1 cm depth in a muscle-tissue equivalent phantom. Devices with heating depths less than 1 cm that are dedicated to use on limited depth superficial disease must fulfill the same criterion at a depth of 0.5 cm. The required temperature increase of 1 °C/min equals a power deposition rate (SAR) of ±60 W/kg [50].

**Fig. 1:**
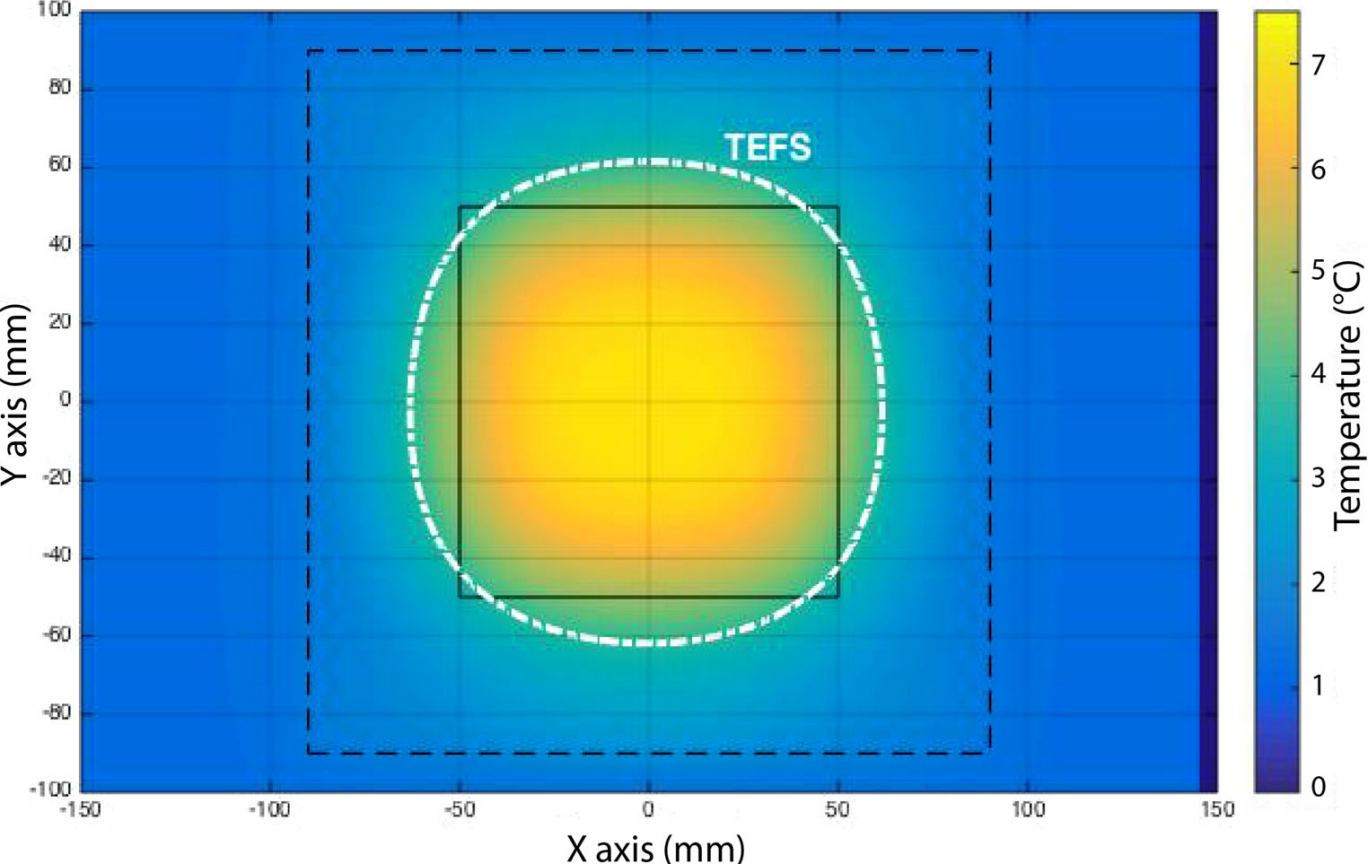
Example of the thermal effective field size (*TEFS*). Calculated normalized temperature rise (*TR*) distribution at 1 cm depth in a two-layered phantom, with fat layer thickness of 10 mm overlying the muscle phantom. Heating time t = 6 min, P = 175 W. The black solid line indicates the applicator aperture, while the black dashed line represents the water bolus. The maximum TR in the 1 cm deep plane in muscle-tissue equivalent phantom is T_max1cm_ = 7.6 °C. The TEFS isotherm then quantifies the area with TR ≥ 3.8 °C

**Fig. 2:**
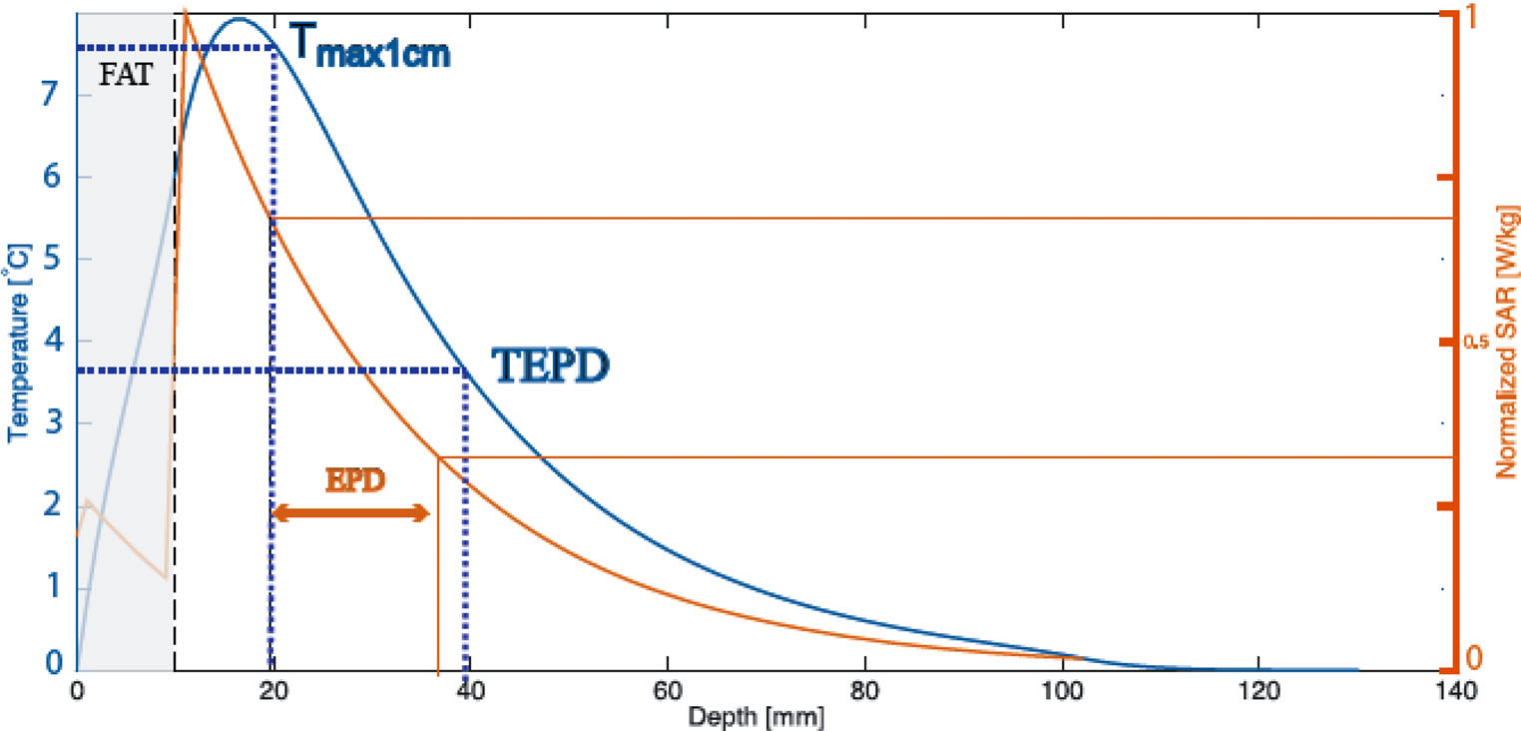
Example of the thermal effective penetration depth (*TEPD*). Simulated temperature increase (*blue – left axis*) and specific absorption rate (*SAR*; *red – right axis*) as a function of depth in the center of two-layered phantom, with fat layer (*blue*) thickness of 10 mm. Heating time t = 6 min with P = 175 W and bolus surface temperature identical to the initial phantom temperature. The maximum temperature rise (*TR*) in the 1 cm deep plane in muscle-tissue equivalent phantom is T_max1cm_ = 7.6 °C. The TEPD is thus the depth where the TR is 3.8 °C, i. e., 39 mm from the tissue surface. The maximum SAR at 1 cm depth in the muscle is approximately 75%. The resulting effective penetration depth (*EPD*) derived according to the traditional definition [7] is indicated by the *arrow*. Observe that the effective heat penetration is essentially at nearly the same depth for both definitions

**Fig. 3:**
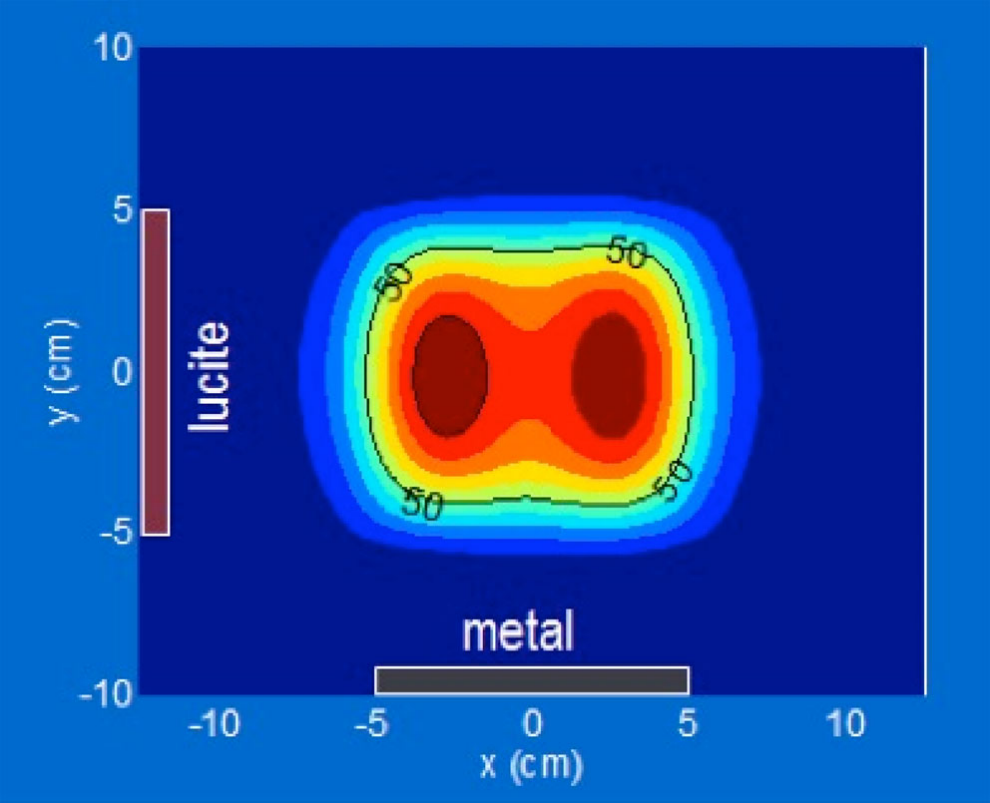
Calculated normalized specific absorption rate (*SAR*) distribution of a 10 × 10 cm lucite cone applicator (*LCA*) at 1 cm depth in a homogeneous muscle tissue phantom. The color scale from *blue* to *dark red* represents a 10% SAR increase for every color transition. Adapted from [66]

The thermal effective field size (TEFS) is defined as the area within the 50% of maximum TR contour (i. e., ∆T ≥ 3 °C) in the 1 cm deep plane under the aperture, see Fig. 1. Similarly, the thermal effective penetration depth (TEPD) is defined as the depth at which the maximum TR is 50% of the maximum TR (i. e., ∆T ≥ 3 °C in 6 min) at 1 cm depth as shown in Fig. 2. Note that the maximum TR at 1 cm depth does not necessarily occur in the central cross section plane through the applicator as illustrated in Fig. 3.

For consistency, the measurements to determine TEFS must be made within 15 s after the end of heating to minimize thermal conduction smearing of the SAR pattern within the phantom. If a multi-element applicator array is used, elements must be arranged such that a continuous region of TR > 50% of the maximum TR exists. This assessment should be made at a depth of 1 cm. In order to determine TEPD, analogous measurements at other depths (>1 cm) are needed until TEPD is found.

Note that besides characterizing the temperature distribution, the efficiency of power transfer within the complete heating system (cables, applicator, and bolus) should be measured. This information can be used as a reference value to compare the clinical applied power levels between hyperthermia centers and assess whether realistic powers are applied to assure the required increase in tumor temperature. However, it is also important that the user is aware of how much power is lost in cables, applicator, and bolus. Efficiency measurements can be accomplished either with a calorimetric^2^ method or the total absorbed power may be calculated from rate of TR measurements.

Note that TR estimation alone is not sufficient for a complete characterization of the system. Numerical modeling and/or experimental assessment in terms of other parameters like SAR are recommended to achieve a more fundamental characterization of the system and to assist the user with determining the relevant parameters for the actual clinical setup. SAR can be determined with the TR measurement procedure described above if a short measurement time of 1 min or less is used to prevent blurring of the SAR pattern by heat conduction. This will require a sufficiently high power output to achieve an adequate temperature rise (e. g., >3 °C) in that short time interval.

The following SAR criteria will then apply for applicator characterization [7]:

- In these guidelines, we recommend deriving both TEFS and TEFD from TR measurements. However, the values derived from SAR, as defined below, may also be useful, e. g., for comparisons with numerical calculations:
- The effective field size (EFS) is defined by the area within the 50% of maximum SAR contour in the 1 cm deep plane under the aperture.
- The effective penetration depth (EPD) is defined by the depth where the SAR falls to 50% of the maximum SAR at 1 cm depth. Note that the maximum SAR may not be in the plane through the main central axes of the applicator.

### Generic phantoms

Two models are necessary to characterize heating of two typical disease conditions: (1) superficial chestwall disease that extends from the tissue surface to moderate depth (generally <15 mm), and (1) tumors at moderate depth underlying normal skin and fat.

For reliable phantom measurements the general rule is that the phantom size should extend beyond the applicator by at least 10 cm in all directions. The thickness of the phantom should be at least three times the expected penetration depth. Applying this rule leads to the following recommendations for phantom dimensions:

- Superficial chestwall disease,
  - *EM-radiative systems:* The block phantom should consist of homogeneous muscle tissue equivalent material at least 10 cm thick and extending laterally 10 cm beyond the physical dimensions of the applicator or 5 cm beyond the bolus, whichever is larger.
  - *EM capacitive systems:* The phantom thickness should be at least 15 cm with lateral dimensions at least twice as large as the electrodes.
  - *IR systems:* The phantom size should extend beyond the expected TEFS by at least 5 cm and should have a thickness of 5 cm.
  - *US systems:* The phantom should consist of homogeneous muscle or “soft tissue” equivalent material extending laterally 5 cm beyond the physical dimensions of the applicator or the bolus, whichever is larger, and the thickness should be at least three times the expected penetration depth.
- Tumors at moderate depth underlying normal skin and fat,
  - *EM radiative systems:* To model tumors underlying normal fat, a 1 cm thick layer of fat tissue-equivalent phantom should be placed on top of the muscle phantom which should then have a thickness of at least 9 cm.
  - *EM capacitive systems:* To model tumors underlying normal fat, a 1 cm thick layer of fat tissue-equivalent phantom should be placed on top of the muscle phantom which should then have a thickness of at least 14 cm.
  - *IR systems:* Similar to EM phantom with fat layer thickness of 0.5 and 1 cm.
  - *US systems:* Similar to EM phantom with muscle phantom thickness at least three times the expected penetration depth.

In all phantoms, the muscle phantom should be composed of at least two layers separated by a thin (≤0.1 mm) polyester, cheesecloth, or mylar film. The US phantoms should be coupled with liquid or gel, and separated by thin <50 um polyethylene or mylar film. The thickness of the upper layer should be 1 cm. For more extensive evaluation of distributions at different depths in the phantom, the block of muscle phantom may be separated into additional layers, with thicknesses of 0.5, 1, 1.5, 2.5 cm in case of EM devices operating at a frequency of 915 MHz or higher and 1, 1.5, 2.5, 4.5 cm for other frequencies. The fat phantom should consist of two layers with thickness of 0.5 cm.

If the power deposition pattern will be characterized as a function of depth with an IR camera, the muscle phantom should be split in two halves along a vertical cross-section along the center of the phantom. The applicator should be positioned with its central axis aligned with the vertical measurement plane of the phantom. The applicator should be rotated 90° between successive measurements to determine the pattern along both central (major and minor) axes of the applicator. A 1 cm thick fat phantom layer split down the middle should be added to the top of the muscle phantom to model tumor underlying normal fat.

For all temperature and power deposition measurements, a proper phantom must be used with the correct electrical, optical, or ultrasound properties and correct thermal properties. Tissue equivalence of the phantom material can be obtained by using freshly made materials following a validated recipe or demonstrating equivalent properties via measurements.

Phantom recipes for electromagnetic equivalent phantoms at commonly used frequencies are specified in Appendix B; a limited set of suggestions of EM–phantom mixtures for less frequently used frequencies, ultrasound phantoms, and infrared phantoms are also provided.

### Test conditions

The thermal distribution of the applicator should be characterized under standardized operating conditions and at all frequencies in clinical use. The two generic reference phantom set-ups described in Section *Generic phantoms* should be used for this characterization.

**Fig. 4:**
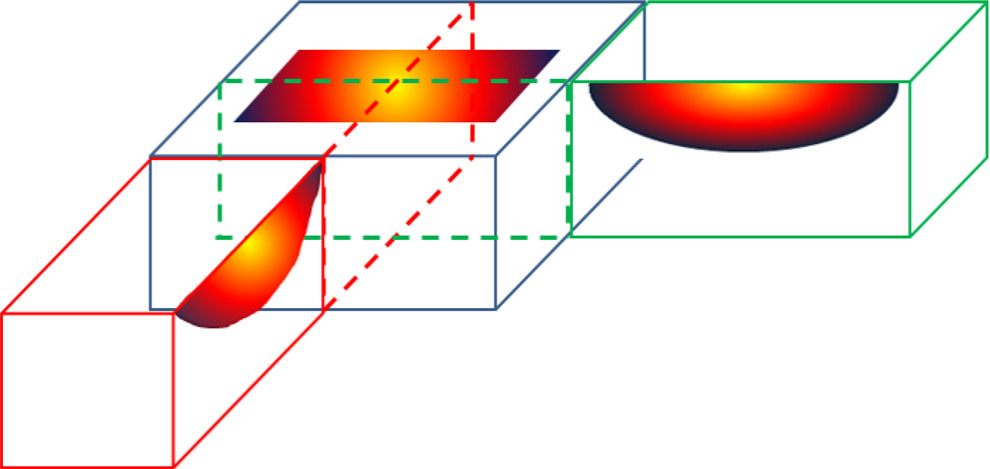
Thermal effective field size (*TEFS*) profiles should be measured in three orthogonal planes crossing the center of the applicator

All measurements should be carried out at room temperature, i. e., the phantom model should be at equilibrium with room temperature at the start of each experiment. A bolus of appropriate dimension should be placed on top of the phantom with liquid circulating at the same room temperature as the rest of phantom. The distribution of temperature rise must be measured in three orthogonal planes crossing the center of applicator as shown in Fig. 4.

The horizontal plane at 1 cm depth must always be measured by an IR camera using a horizontally split phantom with a removable top layer of 1 cm which is removed for the IR temperature measurement.

**Fig. 5:**
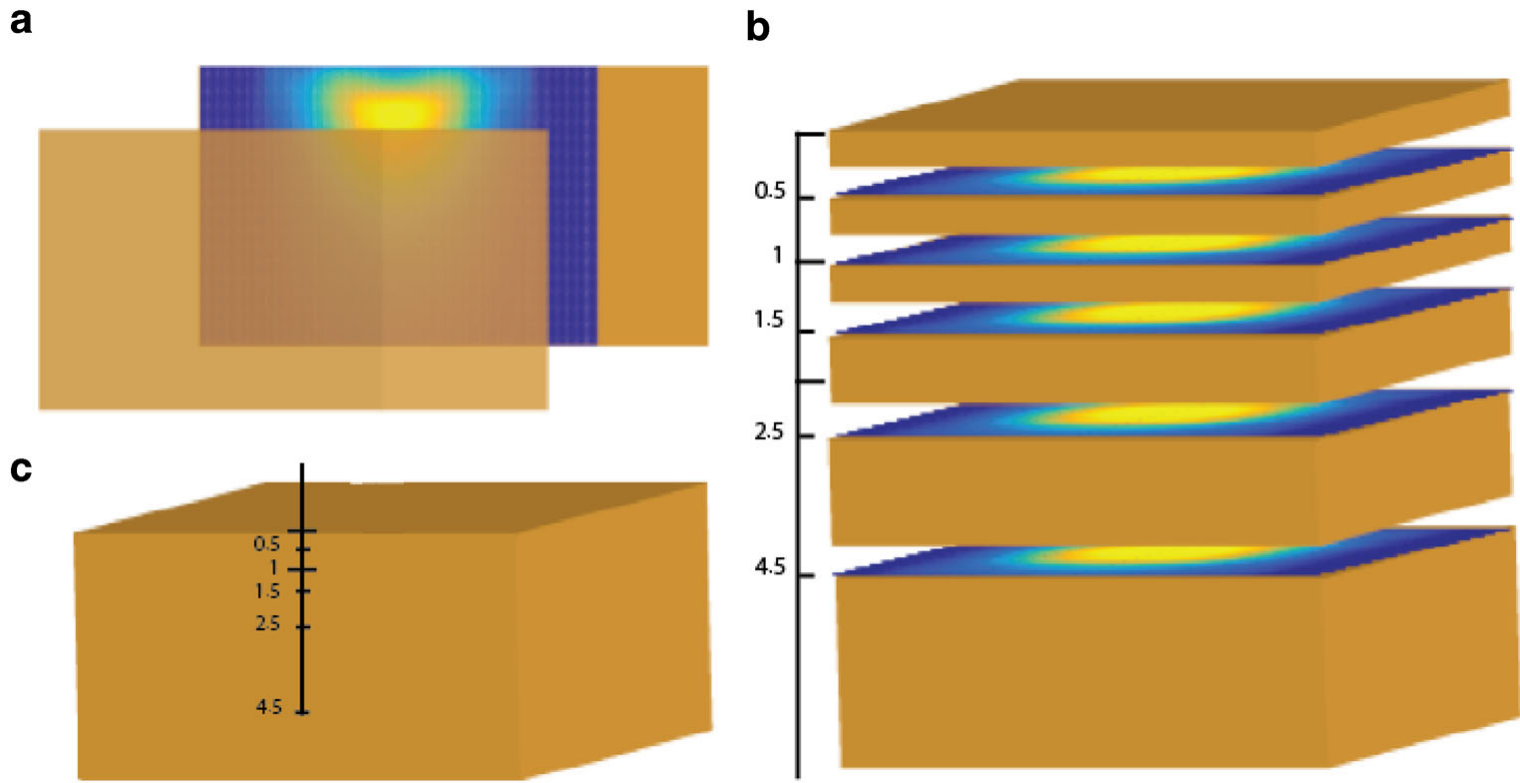
Illustration of three alternative options to obtain thermal profile with depth: **a** thermal camera view of vertical plane containing peak temperature rise (*TR*); **b** reconstruction of vertical distribution from thermal camera views of multiple horizontal planes; and **c** multiple measurements along a single axis depth probe

The vertical planes (xz, yz) can be measured with one of three alternative methods as shown in Fig. 5:

- with IR camera using a vertically split phantom or
- with IR camera in multiple horizontal planes and post processing of data from multiple planes,
- with temperature probes in catheters running through the phantom at the defined depths. Although this method can be used, the ESHO-TC prefers the use of methods (a) or (b) which produce much higher resolution characterization of the distribution.

Alternative method (a): Use of a vertically split phantom is highly recommended for best resolution of TR distribution.

Alternative method (b): To reconstruct the vertical TR distribution from multiple horizontal plane data, the measurement planes must include depths of 0.5 (for ≥915 MHz only), 1, 1.5, 2.5, 4.5 cm and additional depth planes as needed to measure SAR < 10% of max SAR under the applicator.

Alternative method (c): Multiple stationary temperature probes must be used to obtain a minimum resolution of 1 cm. The absolute TR at each depth measured immediately after switching on EM, US, or IR power must be used to plot the temperature depth profile as a means to determine TEPD.

With any of the methods, if the vertical plane measurements show a deviation of the temperature maximum from the central plane, an additional measurement must be performed through the vertical plane along the cross-section of the applicator that includes the true maximum TR. In addition, at least one temperature sensor must be placed at 1 cm depth in the center of the central plane of the applicator as a reference point regardless of the test set-up applied. The absolute TR measured at this location is indicative of the efficiency of the applicator to deposit a high SAR at this position and combined with applicator input power allows simple quantitative comparison between institutes of the temperature increase obtained in the fixed 6 min heating interval. The SAR is related to the temperature increase in six minutes of heating according the simple formula: SAR [W/kg] = 67 × dT/dt [°C/min] [50].

#### Required specifications of the IR thermal imaging

As discussed in method (b), IR thermal imaging is required for characterization of the temperature distribution in the horizontal plane at 1 cm depth in phantom. The resolution of readings and relative accuracy (NETD) of the thermographic camera should be 0.1 °C and 0.05 °C or better, respectively. The spatial resolution should be 1–2 mm for phantoms less than 30 cm^2^, and 2–3 mm for larger phantoms. The camera should be placed at a proper height to cover the entire phantom surface and test images should be recorded with rulers in the field of view to ensure correct rendition of dimensions.

## Disclaimer

This publication is based on literature and other sources of information judged to be reliable by the authors representing the ESHO-TC. However, the authors, ESHO-TC, and editors disclaim any warranty or liability based on or relating to the contents of this publication. The authors and ESHO-TC do not endorse any products, manufacturers, or suppliers. Nothing in this publication should be interpreted as implying such endorsement. Several companies were invited to provide feedback on the document but have not participated actively either at ESHO-TC meetings or in the writing of this document. Some companies were invited to provide feedback on the document but have not participated actively either at ESHO-TC meetings or in the writing of this document. The authors and ESHO-TC alone are responsible for the content and writing of this paper.

## Appendix A

### Detailed description of applicator types with their advantages and limitations

#### EM radiative system

For purposes of the present guidelines, radiative applicator options may be divided into two main types: waveguide applicators and microstrip applicators.

#### Waveguide applicators

Waveguide applicators are made from a section of waveguide transmission line open at one end with an aperture of at least a half wavelength in the direction of the E‑field. The excitation is provided by a short extension of the coaxial feedline center conductor or a loop antenna inside the waveguide structure. In order to reduce the physical dimensions of the aperture, the applicator may be filled with a material having a dielectric constant higher than air, such as Teflon, ceramic, high dielectric powder, or distilled water [12]. A useful subgroup of waveguide applicators includes horn applicators that are made by flaring the opening to spread the radiated field. In one design optimized for use in an array to heat larger tissue volumes, the lucite cone applicator provides an effective heating area approximately 2–3 times larger than a conventional waveguide by using both a dielectric cone in the center of the aperture and a flared horn configuration with two metal walls of the waveguide replaced with dielectric to spread the field [17, 18].

- Advantages:
  - High ratio of E‑field parallel rather than perpendicular to tissue interfaces such as fat layer.
  - Robust applicator with reliable performance.
  - Somewhat deeper penetration from nearly plane wave radiation parallel to the skin surface.
  - Spatial control of SAR when used in multi-applicator configuration.
- Limitations:
  - Bulky applicators require stable mounting and positioning system.
  - Rigid shape does not conform to contoured anatomy; when used in arrays, this limitation is less restrictive.
  - Many waveguide structures do not provide heat coverage >25% SAR_max_ to the periphery of aperture so may have regions of tissue <50% SAR_max_ between adjacent apertures of multi-applicator array.

#### Microstrip applicators

Microstrip applicators generally consist of printed circuit metallic patterns on one side of a thin substrate and backed by a continuous metal ground plane. A water bolus is typically used to couple the applicator to the contoured tissue surface and is often mechanically connected to the sidewalls or back plane of the applicator. Clinically utilized examples are contact flexible microstrip applicator (CFMA) [19–22, 26, 36], Archimedean spiral applicator [27–29], dual concentric conductor (DCC) [30–33], and current sheet applicator (CSA) [25, 34]. Although the radiation patterns from each aperture differ among the various microstrip designs, several common attributes can be generalized: thin, low profile or flexible substrates, small dimensions per radiating structure, and straightforward combination into multiple antenna arrays.

- Advantages:
  - Lightweight and small size, easy to set up.
  - Readily combined into multi-element array applicators.
  - Many of the array applicators can conform to contoured anatomy.
  - Many types have integrated water bolus.
  - Comfortable for the patient.
  - Spatial control when used in multi-element applicator configurations with equal number of RF-power generators.
- Limitations:
  - Some have significant electric field component normal to skin surface and tissue interfaces, which may reduce the penetration depth substantially.
  - Some have power limitations due to potential overheating or high reflected power.

### EM capacitive systems

EM capacitive heating utilizes two electrodes placed on either side of the body to produce superficial hyperthermia in the proximity of one of the two electrodes [52, 53]. In order to heat superficial locations, an electrode matching the lesion size is placed on the target lesion while a larger electrode that spreads out the current density over a larger area is situated on the opposite side of the body. The electrodes are connected to an RF power source (8–27 MHz) and the resulting power deposition pattern is mainly determined by the size and location of the smaller electrode. Descriptions of the use of EM capacitive heating are given in [54, 55]. An important factor to consider when applying EM capacitive heating is to be careful of unintended preferential heating of the high resistance fat tissue layer [56]. Temperature control of the circulating water in the coupling bolus is used to address overheating of superficial fat tissue.

- Advantages:
  - Easy setup.
  - Good penetration depth.
- Limitations:
  - No capability for multi-applicator configuration.
  - Can overheat fat layer relative to underlying tumor target.
  - Unintended high temperatures at the edge of metal electrodes if not coupled properly.

### US heating systems

Ultrasound heating may be accomplished with round or square piezoceramic plates or composite transducers, which create a pressure wave in front of the transducer when excited at their resonant frequency. The traveling pressure wave reflects from air so is generally coupled to tissue with temperature-controlled degassed water and ultrasound gel to minimize air in the path [57, 58]. Tumor size volumes may be heated by combining multiple transducers in a nonfocused planar array for superficial heating [59, 60], or a phased array that creates an intense focal hot spot or shaped distribution that can be electronically or mechanically scanned at depth to heat a tumor [61–65]. Further information can be found in a recent review [14].

- Advantages:
  - More directional than EM heating, easily focused into tumor with minimal “sidelobes”.
  - Planar array provides more precisely adjustable SAR patterns that conform to the transducer geometry.
  - Thickness of water bolus is mostly irrelevant since degassed water is not lossy.
  - Minimal reflections from the edges of water bolus as long as bolus extends beyond the transducer perimeter.
  - Good penetration depth.
- Limitations:
  - Preferential absorption of ultrasound in bone and therefore risk of pain when the tumor is located close to or overlying bone.
  - Reflections and/or absorption of ultrasound at tissue interfaces with fat, bone, or air/gas (nearly 100%) inside the body.
  - Must degas the water by vacuum extraction or boiling to minimize transmission losses through water bolus.

### IR devices

IR radiation, also known as thermal radiation, is divided according to its wavelength into three bands (IR-A, IR-B, IR-C). For the longer wavelengths (IR-C), most energy is absorbed in the skin surface. Therefore the IR-A radiation associated with wavelengths between 780–1400 nm is useful for heating superficial cancers [15, 67, 68, 70]. Due to heat dissipation by conduction, convection and Mie-scattering (forward scattering) the primary absorbed radiation energy is deposited within a larger tissue volume than the original column of absorption. In order to minimize thermal exposure of the skin surface, a water filter system must be integrated with the IR radiation source (wIRA) [15]. The effective penetration depth of 15 mm has been reported for such a system [69]. Surface temperatures of T ≥ 42 °C can be achieved with this heating approach within 5–10 min. The heating pattern can be shaped using an opaque mask to block IR radiation at the locations where it is not needed.

- Advantages:
  - Heating without contact to patient surface achieves conformal treatment without potentially painful patient contact.
  - wIRA radiator controlled by noninvasive high-resolution feedback from thermographic camera adjacent to radiator.
  - Power deposition pattern may be adjusted by placing shielding sheets under the wIRA radiator to completely block nontarget tissues.
- Limitations:
  - Effective heating depth limited to approximately 15 mm.
  - Heating adjustment using 100% blocking sheets is primarily on or off rather than variable heating power adjustment across large surface area.

**Table A.1:** General performance of heating systems

		Thermal effective penetration depth	Dealing with problems at the interfaces^a^	Spatial power control		Thermal effective field size	Ease of use and positioning	Comfort to the patient	Temperature control	Commercial
				Dimensions	Resolution					
EM waveguide	Single applicator	+	++	1D	–	- to +	0	0	+	yes
	Array	+	++	2D	++	0 to ++	-	0	++	no
EM microstrip	Single applicator	0	++	1D	–	0	+	+	+	yes
	Array	+	++	2D	++	++	+ to ++	+	++	yes
EM capacitive		+	–	1D	–	+	++	+	–	yes
US	Single applicator	++	0	1D	–	-	0	+	+	no
	Array	++	0	3D	++	-	+	+	++	no
IR		–	++	2D	+	++	++	++	+	yes

The performance is indicated by following grades: ++ very good, + good, o fair, - poor, – very poor

^a^Dealing with the problems at the interfaces (fat layer under the skin and/or muscle/bone interface)

## Appendix B

### Phantom recipes

This appendix identifies recommended phantom mixtures for muscle and fat tissue equivalent phantoms. The recipes are given for EM equivalent phantoms at commonly used frequencies while the US and IR users are referred to specific reports. Use of other phantom recipes is permitted if dielectric, acoustic propagation, or optical properties of the phantom are demonstrated to be equivalent to the muscle/fat tissues at the frequency of the device under test. The literature values of the relevant properties of muscle and fat for commonly used frequencies are summarized in Table B.1 [71–74]. The relevant optical properties, the absorption coefficient and reduced scattering coefficients are not stated in the table, due to their extreme variability with frequency. Data on the optimal IR frequency band for hyperthermia purposes are not available presently and therefore IR users are referred to comprehensive reviews [75, 76].

The list of suggested phantom recipes will be updated on the ESHO webpage [2].

**Table B.1:** Literature values of relevant dielectric [71, 74], acoustic [72, 74], and thermal properties [74] of muscle and fat

		US	RF/microwave				Physical
		2–8 MHz	13.56 MHz	27 MHz	433 MHz	915 MHz	
Muscle	ε_r_ [−]	–	138	95.9	56.87	55.00	–
	σ [S/m]	–	0.628	0.654	0.81	0.95	–
	α/f [dB/cm/MHz]	1.1	–	–	–	–	–
	c [m/s]	1588.4	–	–	–	–	–
	ρ[kg/m^3^]	–	–	–	–	–	1090
	c_p_ [J/kg/K]	–	–	–	–	–	3421
	k [W/m/K]	–	–	–	–	–	0.49
Fat	ε_r _[−]	–	25.4	18	11.6	11.3	–
	σ [S/m]	–	0.055	0.06	0.08	0.11	–
	α/f [dB/cm/MHz]	0.6	–	–	–	–	–
	c [m/s]	1440.2	–	–	–	–	–
	ρ[kg/m^3^]	–	–	–	–	–	911
	c_p _[J/kg/K]	–	–	–	–	–	2348
	k [W/m/K]	–	–	–	–	–	0.21

Where *ε*_r_ [−] is relative dielectric constant, *σ* [S/m] is electric conductivity,* α/f* [dB/cm/MHz] is attenuation per frequency, *c* [m/s] speed of sound,* ρ* [kg/m^3^] is material density, *c*_p_ is specific heat capacity [J/kg/K], *k* is thermal conductivity [W/m/K]

### EM phantoms

#### Muscle equivalent phantoms

Among numerous recipes for muscle tissue-equivalent phantoms, two alternative formulas are recommended due to their appropriate electrical and mechanical properties, homogeneity, and simplicity of preparation. The ingredients are listed in Table B.2. The agar phantom originally suggested by Nilsson [77] includes NaCl to adjust conductivity and sugar to provide appropriate permittivity. This phantom has the advantage of simple preparation that facilitates reliable phantom homogeneity. The mechanical strength of the phantom can be adjusted by the addition of TX-150/151^3^ gelling powder, also known as Superstuff [78]. Further, the TX-150/151 increases viscosity and, therefore, allows addition of polyethylene powder to lower the permittivity to that of tissue. Both phantoms are relatively easy to prepare, though the later requires more experienced users to ensure homogeneity. The lifetime of both phantoms is limited to a few weeks due to loss of moisture and growth of mold within the phantom. For phantoms for capacitive systems a deviation in permittivity is inevitable which justifies selecting the simple agar and NaCl solution phantom. For a representative electric conductivity it is recommended to use 3.3 g NaCl per liter phantom, giving an estimated value of 0.6 S/m.

#### The agar phantom preparation

1. Dissolve NaCl in deionized water.
2. Add the sugar and heat the solution to around 50–60 °C in order to dissolve it properly.
3. Add the agar slowly while stirring and heat the mixture to the boiling point (80–90 °C for about 5 min).
4. Pour slowly into the container or mold for cooling and solidifying. The mixture solidifies at room temperature within 24 h. Placement of container in cold water bath will speed up the cooling process.

#### The superstuff–agar phantom preparation

1. Dissolve NaCl in deionized water.
2. In a separate container, mix the TX-150 (or TX-151) with the polyethylene powder.
3. Heat the solution of NaCl and deionized water to around 70 °C, and at that point, slowly add the agar while stirring. Continue stirring until the agar is completely dissolved. When the mixture is up to 90 °C, remove it from the heat.
4. When the solution has a temperature of 70–80 °C, add the mixture of TX-150 (or TX-151) – polyethylene powder while stirring continuously to ensure homogeneity. Be careful to avoid generation of air bubbles due to overvigorous stirring, as this will reduce phantom homogeneity. During the process of adding the mixture, keep the temperature of the solution between 70–80 °C. It might be necessary to turn on/off the heater from time to time.
5. When all the mixture has been added, turn off the heater.
6. If formaldehyde is used to slow the growth of mold, carefully add it in this stage.
7. Continue stirring until the phantom partially hardens which is essential to keep the polyethylene powder uniformly mixed.
8. Pour slowly into the container or mold for cooling and solidifying. The mixture solidifies at room temperature within 24 h. Placement of the container in cold water bath will speed up the cooling process. Experience is necessary to optimize the time of transfer from the mixing container to phantom mold. If poured when the mixture is not sufficiently gelled, the polyethylene powder will partially separate. If the phantom gets too stiff in the mixing container, when phantom transferred to the mold and pushed to fill the corners, large air pockets may become trapped inside the phantom producing heterogeneous phantom properties.

**Table B.2:** Muscle phantom recipes. The quantities are given for preparation of 1 l of phantom

	13 & 27 MHz	433 MHz	915 MHz	433 MHz	915 MHz
Reference	[79]	Adjusted recipe of [78]		Adjusted recipe of [77]	
TX 150^b^/TX 151 (Superstuff)	–	19.3 g	18.3 g	–	
Agar powder	40 g	20.9 g	20.9 g	16 g	16 g
Formaldehyde 8%	–	6.7 g	6.7 g	–	–
NaCl (salt)	2.4 g	3.8 g	4.8 g	10 g	7 g
Deionized Water	≈956 g	701.6 g	701.6 g	570 g	570 g
Sugar	–	–		400 g	447 g
Polyethylene powder	–	50.1 g	57.6 g	–	
Permittivity	79.0	60.7	52.8	58.7	52.4
Conductivity S/m	0.44–0.6^a^	0.85	1.07	0.67	0.98

^a^The lower value is recently measured, whereas the higher value is taken from the literature [79]. The difference can be explained by the absence of highly toxic preservative, NaN_3_. Hence, it is recommended to use 3.3 g NaCl per liter phantom to achieve an estimated value of 0.6 S/m

^b^TX 150 & TX 151 (Oil Center Research, Lafayette, LA, USA); * In the original work [70], highly toxic NaN_3_ was used as preservative. Other, less toxic preservatives were given with the mass equivalent quantities of NaCl, to compensate the conductivity decrease. Here, the recipe is adopted for use with formaldehyde 8%. The phantom can be however prepared even without this preservative. In such a case, an equivalent amount of water should be used.

#### Fat equivalent phantoms

An optimal phantom recipe representing properties of fat tissue is currently not available. A base formula for good phantom representing fat tissue in frequency range 10 MHz to 1 GHz was proposed by Guy et al. [51] and later adapted by Balzano et al. [81] for 30 MHz. The material consisting of Laminac 4110 (a polyester resin), acetylene black, and aluminum powder offers great stability and long shelf life; nevertheless, it requires relatively complex preparation. An alternative, easy to prepare formula, proposed by Lagendijk and Nilson [82], is mixture of flour, oil, and saline water. It provides homogeneous phantom with appropriate electrical and mechanical properties; however, the life is relatively short due to (easy) water evaporation the phantom should be covered by thin plastic foil and stored in a ‘closed’ environment. The phantom was reported for frequency 434 MHz but can be easily adopted for other frequencies. The recipes for fat-equivalent phantoms and the corresponding references are listed in Table B.3. Yet another option is use of pig fat, which is easily accessible and has dielectric properties comparable to human fat.

**Table B.3:** Fat tissue equivalent phantom recipes

	13 & 27 MHz	433 MHz	915 MHz		433 MHz	915 MHz
Reference	[81]	[22, 80]			[82]	
*Laminac 4110*	79%	69.30 g		85.2%	–	
*Aluminum powder*	20.72%	29.46 g		14.5%	–	
*Shawinigan/Acetylene black*	0.28%	0.30 g		0.24%	–	
*XC-72 Carbon powder*	–	0.94 g		–	–	
*MEK peroxide/*P *102 resin*	7cc/2 kg	*I part per 200 parts resin*		0.375%		
Flour	–	–			500	
Oil	–	–			225	
Saline *(0.9% NaCl per liter)*	–	–			25	
Dielectric constant	19	5.5	5.61		7.1	9.1
Conductivity/S/m	0.028	0.04	0.0665		0.11	0.16

### US phantoms

#### Muscle phantom

The recommended muscle or “soft tissue” mimicking phantom for acoustic and thermal testing of ultrasound thermal therapy devices was developed recently by investigators at the US Food and Drug Administration [83]. This nontoxic formulation has thermal conductivity, acoustic attenuation, and acoustic impedance similar to soft tissue. The recipe utilizes a gellan gum as gelling agent with melting temperature above 100 °C, calcium chloride dehydrate to enhance the gel strength and potassium sorbate which acts as preservative. The ultrasound attenuation is simulated by using different sizes of aluminum oxide particles, while the acoustic velocity is adjusted by isopropanol. The protocol and complete recipe can be found in the literature [84]. The attenuation coefficient has essentially linear frequency dependence, as is the case for most mammalian tissues at 37 °C. The mean value of attenuation coefficient is 0.64*f^0.95^ dB cm^−1^ at 20 °C in the measured frequency range from 2 to 8 MHz. When using low frequency ultrasound sources, penetration through the phantom can be high so the thickness of phantom model needs to be sufficient to fully absorb the energy prior to reflections off the bottom of phantom. Alternatively, an absorber such as a soft 20–30 durometer rubber can be added as a back layer to minimize reflections.

#### Fat phantom

A fat-mimicking phantom in which oil droplets are dispersed in a water-based gelatin [85] is recommended. The phantom has appropriate ultrasonic parameters for fat, such as lower density, speed of sound, and an attenuation coefficient that is nearly proportional to the frequency. The only drawback is the somewhat complex preparation procedure described in [85]. In some cases with split phantoms, where the temperature index (TEFS or SAR) is measured in the deeper solid soft tissue layer, it may be possible to use a suspension of pure safflower oil and polyurethane mesh within a shaped bag and of appropriate thickness to mimic the acoustic impedance and penetration characteristics of an intervening layer of fat [86].

### IR phantoms

Due to the extreme variability of dielectric properties over the IR frequency spectrum, it is only possible to define phantoms for very specific portions of the spectrum. IR users are, therefore, referred to a recent review [87] which discuss the benefits and weaknesses of various recipes (for optical spectroscopy, imaging and dosimetry) in terms of gelling agents and materials that mimic scattering and absorption. Two alternative compositions can be recommended: (1) The agar phantom with Intralipid™ acting as a scattering medium, and India ink acting as an absorbing medium [88]. Note that agar might affect the optical properties and therefore only highly purified powder can be used. (2) A silicon-based phantom [89] whose absorption and scattering properties are adjusted by adding matrix cosmetic powder and Al_2_O_3_ particles to the silicone base. This phantom exhibits good macroscopic homogeneity can be cast into layers or arbitrary shapes with long shelf life.

## Appendix C

### Routine QA for superficial microwave hyperthermia system

During routine use, a deterioration of applicator performance could occur. It is typically indicated by a sudden change in the power required to achieve the desired temperature. The operator should be continuously alert for such changes. As part of a clinical quality assurance program, general QA routines common to most hyperthermia systems are specified in Table C.1. These should be performed at regular intervals as a part of the clinical QA program. System specific QA procedures given by manufacturers/suppliers will be available on the ESHO website [2].

**Table C.1:** Routine quality assurance procedures

Interval	Daily		Quarterly 3 months		6–12 months	
Topic	Visual check	Acceptance criterion	Check function	Acceptance criterion	Check function	Acceptance criterion
Temperature probes	Broken probes	Accuracy ±0.2 °C	Full range calibration	Accuracy ±0.2 °C over 37–45 °C	–	–
Water bolus	Leakage, intact structure	Good circulation	–	–	–	–
Water system	Tubing and connections	Good circulation	Water exchange	Correct electrical conductivity	–	–
Power system	Damage	Power output as expected	–	–	Full calibration, power output	Power reading forward and reflected ±5%
Applicators	Damage	SAR pattern as expected	–	–	Heating pattern	TEFS, TEFD, EFS, efficiency
Control system	Interaction of commands	Proper functioning control algorithm	Back-up system	Proper function	Update software	Verify system performance
Safety	Cable connections	Correct undamaged connections	–	–	Stray radiation	Values within EU/FCC regulations

## Footnotes

1 This range includes room temperature as required for QA, in addition to the 30–45 °C range typical for clinical treatments.

2 The efficiency of the superficial hyperthermia system is obtained by calculating the ratio of the EM power absorbed in the phantom to the net power input to the applicator (forward minus reflected power measured at the output of the amplifier minus cable losses between amplifier and applicator). The EM power absorbed in the phantom is measured using the calorimetric method: a well-insulated and well-stirred liquid muscle-tissue equivalent phantom is heated by the applicator for 10 min with a high EM-power input. The homogeneous temperature increase of the liquid muscle-tissue equivalent phantom is measured and total absorbed energy calculated from P_a_ = ρ∗c_p_∗V∗dT/dt [J/s], in which P_a_ is absorbed power, ρ is the density, and c_p_ is the specific heat capacity of the liquid muscle-tissue equivalent phantom material, V is the volume, dT the temperature increase, dt is the duration of heating.
